# Fat Chance: The risk of Vortioxetine-induced Hypertriglyceridaemia

**DOI:** 10.1192/j.eurpsy.2025.1306

**Published:** 2025-08-26

**Authors:** E. Cahalan

**Affiliations:** 1 Psychiatry, HSE, Galway, Ireland

## Abstract

**Introduction:**

Hypertriglyceridaemia as a side effect of antidepressants including venlafaxine and mirtazepine has been described in the literature. A relatively new SSRI, vortioxetine has never been reported to contribute to hypertriglyeridaemia and it is not mentioned in the prescribing information for this agent. Dyslipidaemia is an important risk factor for morbidity in people who suffer with mental illness, most often observed in patients who are prescribed antipsychotic medications. Evidence to date with antidepressant medication is mixed but may represent a clinically relevant additional cardiovascular risk.

**Objectives:**

This case report explores the possible relationship between Vortioxetine and worsening hypertriglyceridaemia in a patient with depressive illness.

**Methods:**

Clinical case report and brief literature review

**Results:**

A 58 year old female, with a history of Recurrent Depressive Disorder was admitted to an acute psychiatric inpatient unit with a relapse in depressive illness with anxious distress and suicidality. Prior to admission, her medications included Mirtazepine 60mg, Duloxetine 120mg and Olanzapine 7.5mg. She had a family history of dyslipidaemia. A blood panel was taken which revealed mildly deranged lipid profile - Total Cholesterol of 7.72mmol/L (Upper limit of Normal - 5.0mmol/L); HDL 1.02mmol/L (ULN - 2.0mmol/L); LDL 5.44mmol/L (ULN - 3.0mmol/L); Triglycerides 2.78mmol/L (ULN - 2.0mmol/L).

The patient’s Duloxetine was switched to Venlafaxine and repeat lipid levels one week later revealed a dramatic rise in triglycerides to 12.16 mmol/L, and then 14.07 mmol/L 2 days later again. Venlafaxine was discontinued in view of these findings with a reduction in serum triglycerides to 11.43mmol/L within 24h.

Vortioxetine 10mg was commenced and the patient’s lipid profile was again repeated with an upswing in triglyceride levels to a peak of 15.4mmol/L within 48h of commencement. Vortioxetine was discontinued and fenofibrate was commenced to treat the patient’s dyslipidaemia due to a risk of acute pancreatitis and atherosclerotic disease. Serum triglycerides reduced to near-normal levels within days.

**Conclusions:**

This case report highlights the risk of dyslipidaemia associated with antidepressant prescribing and identifies vortioxetine as an agent which may contribute to this. This patient had a number of risk factors for developing hyperlipidaemia including co-prescription with olanzapine and mirtazepine which have known metabolic effects, as well as a family history for hyperlipidaemia and a post-menopausal state. However, it was notable that her significant hypertriglyceridaemia was cause by venlafaxine, and exacerbated further by vortioxetine. Cardiovascular risk profiles must always be considered in the psychiatric patient population and we feel that this case report may add to the literature around commonly prescribed antidepressant medications and their contribution to this risk.

**Disclosure of Interest:**

None Declared

